# Treatment with adipose tissue-derived mesenchymal stem cells exerts anti-diabetic effects, improves long-term complications, and attenuates inflammation in type 2 diabetic rats

**DOI:** 10.1186/s13287-019-1474-8

**Published:** 2019-11-20

**Authors:** Songyan Yu, Yu Cheng, Linxi Zhang, Yaqi Yin, Jing Xue, Bing Li, Zhengyuan Gong, Jieqing Gao, Yiming Mu

**Affiliations:** 1 School of Medicine, Nankai University, Tianjin, China; 2 Department of Endocrinology, Chinese PLA General Hospital, Medical School of Chinese PLA, Beijing, China; 3 Department of Endocrinology and Metabolism, Peking University Third Hospital, Beijing, China

**Keywords:** Diabetes, Diabetes complications, Mesenchymal stem cells, Macrophage, Inflammation

## Abstract

**Background:**

Long-term diabetes-associated complications are the major causes of morbidity and mortality in individuals with diabetes. These diabetic complications are closely linked to immune system activation along with chronic, non-resolving inflammation, but therapies to directly reverse these complications are still not available. Our previous study demonstrated that mesenchymal stem cells (MSCs) attenuated chronic inflammation in type 2 diabetes mellitus (T2DM), resulting in improved insulin sensitivity and islet function. Therefore, we speculated that MSCs might exert anti-inflammatory effects and promote the reversal of diabetes-induced kidney, liver, lung, heart, and lens diseases in T2DM rats.

**Methods:**

We induced a long-term T2DM complication rat model by using a combination of a low dose of streptozotocin (STZ) with a high-fat diet (HFD) for 32 weeks. Adipose-derived mesenchymal stem cells (ADSCs) were systemically administered once a week for 24 weeks. Then, we investigated the role of ADSCs in modulating the progress of long-term diabetic complications.

**Results:**

Multiple infusions of ADSCs attenuated chronic kidney disease (CKD), nonalcoholic steatohepatitis (NASH), lung fibrosis, and cataracts; improved cardiac function; and lowered serum lipid levels in T2DM rats. Moreover, the levels of inflammatory cytokines in the serum of each animal group revealed that ADSC infusions were able to not only inhibit pro-inflammatory cytokines IL-6, IL-1β, and TNF-α expression but also increase anti-inflammatory cytokine IL-10 systematically. Additionally, MSCs reduced the number of iNOS(+) M1 macrophages and restored the number of CD163(+) M2 macrophages.

**Conclusions:**

Multiple intravenous infusions of ADSCs produced significant protective effects against long-term T2DM complications by alleviating inflammation and promoting tissue repair. The present study suggests ADSCs may be a novel, alternative cell therapy for long-term diabetic complications.

## Background

Type 2 diabetes mellitus (T2DM) is a long-term metabolic disorder that represents a global public health challenge; not only does T2DM affect industrialized countries, but its impact is also increasing drastically in developing nations [1]. Importantly, T2DM is closely associated with the long-term damage, dysfunction, and failure of various organs, such as chronic kidney failure (CKD), nonalcoholic steatohepatitis, pulmonary fibrosis, cardiovascular disease, and cataracts. Diabetic nephropathy (DN), occurring in 25–40% of individuals with T2DM, has become the single most common cause of end-stage renal disease (ESRD) in the USA, accounting for > 50% of new cases of renal failure [2]. Notably, individuals with diabetes who develop ESRD have a poor prognosis because of a high risk of cardiovascular events [3]. Diabetic cardiomyopathy (DCM) is one of the major causes of mortality and morbidity in diabetic patients [4]. Currently, the first-line treatments widely prescribed for diabetic complications provide only palliative relief but no definitive cure. Hence, an effective strategy to reverse the long-term complications in these patients needs urgent investigation.

Chronic low-grade inflammation, recently referred to as ‘metaflammation’, is thought to be a relevant factor contributing to the development of diabetic complications [5–8]. These immunological changes include alterations in the levels of inflammatory cytokines and chemokines [9, 10], changes in the number of infiltrating leukocytes [11–13], and the development of tissue fibrosis [14, 15]. In clinical studies, the levels of inflammatory markers appear to predict the onset and progression of diabetic complications [9]. It is now generally accepted that tissue-resident macrophages play major roles in the regulation of tissue inflammation. Macrophages exhibit a phenotypic range that is intermediate between two extremes, M1 (pro-inflammatory) and M2 (anti-inflammatory). Treatment with immunosuppressants such as mycophenolate mofetil or sirolimus reduces renal inflammation in association with prevention of the development of glomerular injury in diabetic rats [16, 17]. Similarly, blockade of the MCP-1 receptor (CCR-2) with a selective antagonist suppresses the infiltration of interstitial macrophages and ameliorates diabetic glomerular sclerosis [18]. The results of recent studies highlight the possibility that immunomodulation and, specifically, immunoresolvents are novel strategies to overcome several diabetic complications simultaneously.

Mesenchymal stem cells (MSCs) are fibroblast-like stem cells with the ability to self-renew and undergo multilineage differentiation. Recently, more attention has been paid to the immunomodulatory and anti-inflammatory effects of MSCs [19, 20]. Clinical studies have shown that an infusion of MSCs suppressed systemic inflammation in T2DM patients [21], and MSCs regulated inflammation and promoted repair of damaged tissues in inflammatory diseases such as graft-versus-host disease (GvHD) [22] and systemic lupus erythaematosus (SLE) [23]. While the efficacy of MSC to ameliorate the progression of early-stage diabetic complications is relatively well established, the therapeutic effects of MSCs on advanced diabetic complications are still unknown. Furthermore, there is limited detailed information confirmed by long-term studies on the efficacy and feasibility of multiple intravenous MSC infusions for T2DM with advanced complications.

In this study, we induce a rat model to closely mimic the long-term complications occurred in T2DM. We reported that multiple intravenous adipose-derived mesenchymal stem cell (ADSC) infusions produced significant anti-diabetic effects and hindered the progression of long-term diabetic complications, such as lung, liver, kidney, and cardiovascular complications. Moreover, ADSCs attenuated systemic inflammation and altered the tissue M1/M2 ratio, demonstrating the therapeutic potential of ADSCs on long-term diabetic complications.

## Methods

### Isolation and identification of adipose-derived MSCs

ADSCs were isolated, purified, and identified as described previously [24]. Male Sprague–Dawley (SD) rats weighing 80–100 g were selected and anaesthetized by an intraperitoneal injection of 2% sodium pentobarbital (40 mg/kg). After disinfection with 75% alcohol, the skin of the rats was cut along the abdominal line, and the subcutaneous groin fat was removed. Adipose tissue was washed three times with sterile phosphate buffer solution (PBS) and minced. The extracellular matrix (ECM) was digested with 0.1% type I collagenase and 0.05% trypsin (Gibco, Grand Island, NY, USA) and centrifuged at 1500 rpm for 10 min. The sediment was suspended with low-glucose Dulbecco’s modified Eagle’s medium (DMEM; Gibco) containing 10% foetal bovine serum (FBS; Gibco) and 1% penicillin–streptomycin (Gibco), then transferred into a dish and cultured at 37 °C in 5% CO_2_. After 48 h, no adherent cells were removed, the media were replaced, and the culture medium was replaced twice a week. Passage 3 of the ADSCs was used and phenotyped.

### Animal experiment and treatment

Eight-week-old male SD rats were provided a normal chow diet (NCD) or a high-fat diet (HFD; 60% fat, Research Diets, New Brunswick, NJ) for 8 weeks. After 8 weeks’ HFD, a single dose of 25 mg/kg streptozotocin (STZ) (Sigma-Aldrich, Saint Louis, MO) dissolved in 10 mmol/l citrate buffer (pH 4.5) was intraperitoneally injected to HFD-fed rats. Random glucose was consecutively measured by monitoring tail capillary blood glucose levels. Rats with more than three random glucose level measurements ≥ 16.7 mmol/l were considered diabetic. Then, to generate a long-term T2DM complication rat model, T2DM rats were fed a HFD for 24 more weeks.

To evaluate this model, HFD-fed rats were randomly selected for sacrifice at the time point before HFD (HFD-b), before STZ injection (STZ-b), 1 week after STZ injection (STZ-1w), 12 weeks after STZ injection (STZ-12w), and 24 weeks after STZ injection (STZ-24w). The epididymal adipose, pancreas, and kidney were stained with haematoxylin and eosin (H&E), and body weight, fasting blood glucose (FBG), fasting blood insulin (FBI), serum C-peptide, and urinary albumin to creatinine ratio (ACR) were detected.

At 24 weeks after STZ injection, the T2DM rats were randomly treated through the tail vein with a single infusion of 3 × 10^6^ ADSCs suspended in 0.5 ml of PBS once a week for 24 weeks (referred to as the MSC-treated group) or an infusion of 0.5 ml PBS alone (referred to as the T2DM group) once a week for 24 weeks. On week 57, intraperitoneal glucose tolerance tests (IPGTTs), insulin tolerance tests (IPITTs), and hyperinsulinaemic–euglycaemic clamp study were performed as previously described to assess the therapeutic effects of ADSCs. Whole blood was collected from the left ventricle, and plasma was obtained after centrifugation at 3000 rpm for 10 min. FBG and FBI were detected. Blood lipids (including total cholesterol [TC], low-density lipoprotein cholesterol [LDL-C], and triglyceride [TG]), hepatic enzymes (including alanine aminotransferase [ALT] and aspartate aminotransferase [AST]), serum creatinine, blood urea nitrogen (BUN), ACR, and blood cell counts were measured by the Servicebio corporation.

Homeostatic model assessment index of insulin resistance (HOMA-IR) and of β cells (HOMA-β) was calculated as follows: HOMA-IR index = (FBG, mmol/l) × (FBI, mU/l)/22.5 and HOMA-β index = 20 × (FBI, mU/l)/[(FBG, mmol/l) − 3.5)].

All animal procedures were reviewed and approved by the Institutional Animal Care and Use Committee of the Chinese People’s Liberation Army (PLA) general hospital.

### ADSCs’ homing efficiency in different tissues of T2DM rats

ADSCs were labelled with chloromethyl-benzamidodialkylcarbocyanine (CM-Dil; Life technologies, Eugene, OR, USA) according to the manufacturer’s instructions. Twenty-four weeks after STZ injection, the T2DM rats were infused with ADSCs-Dil (3 × 10^6^ ADSCs-Dil suspended in 0.5 ml PBS). Twenty-four hours and 7 days after ADSCs-Dil infusion, the T2DM rats were sacrificed and their adipose, pancreas, kidney, liver, lung, and heart were removed for preparation of sections. Labelled ADSCs were calculated under a laser scanning confocal microscope (Leica, Wetzlar, Germany).

### Histology and immunofluorescence

For histological analysis, epididymal adipose, pancreas, kidney, lung, cardiac, and lens tissues were fixed in formalin and then embedded with paraffin. Tissues were sectioned into 4–6-μm slices and stained with haematoxylin and eosin (H&E), periodic acid-Schiff (PAS), Masson’s trichrome, Sirius Red, and Oil Red O according to the standard protocol. The morphological structure of each tissue sample was observed under a light microscope, and photomicrographs were taken (Olympus, Japan).

Kidney: The severity of the kidney injury includes infiltration of polymorphonuclear cells, congestion, desquamation, loss of microvilli, and swelling of tubule cells. Kidney injury was graded as follows for each criterion: 0, normal; 1, mild; 2, moderate; and 3, severe. Tubular changes including hydropic degeneration (swelling/vacuolization), desquamation, brush border loss, and peritubular infiltration were graded as follows with a maximum score of 12: 0, normal; 1, mild; 2, moderate; and 3, severe. Glomerulosclerotic injury was graded using PAS-stained sections as follows: 0, normal; 1, ≤ 25% of the glomerular area (mild sclerosis); 2, 25–50% of the glomerular area (moderate sclerosis); and 3, ≥ 75% of the glomerular area (severe sclerosis) [25].

Liver: Steatosis was assessed by Kleiner et al. [26] grading the percentage of steatotic hepatocyte involvement as follows: grade 0, < 5%; grade 1, 5–33%; grade 2, > 33–66%; and grade 3, > 66%. In addition, Brunt’s histological scoring system was used to evaluate the degree of hepatocellular ballooning and lobular inflammation (grade of activity) as well as the stage of fibrosis [27]. Minimal criteria for the histological diagnosis of definite nonalcoholic steatohepatitis (NASH) included the combined presence of grade 1 steatosis, hepatocyte ballooning, and lobular inflammation with or without fibrosis.

Lung: The degree of lung injury degree was evaluated according to Mikawa’s scoring standards [28]: (1) alveolar congestion, (2) haemorrhage, (3) infiltration or aggregation of neutrophils in the airspace or vessel wall, and (4) thickness of the alveolar wall/hyaline membrane formation. Each item was scored on a 5-point scale: 0, minimal damage; 1, mild damage; 2, moderate damage; 3, severe damage; and 4, maximal damage. The final lung injury score was the sum of the four items. Tissue sections were stained with Masson’s trichrome for the evaluation of fibrosis. Glycogen content in type II pneumocytes was graded using PAS-stained sections as follows: normal, not detectable; mild, < 25% of alveolar epithelial cells; mild to moderate, 25–50% of alveolar epithelial cells; moderate to severe, 50–75% of alveolar epithelial cells; and severe, 75–100% of alveolar epithelial cells [29].

For immunofluorescence analysis, tissues were cut into 6-μm sections. Frozen sections were incubated for 14 h at 4 °C with primary antibodies for insulin (1:200, guinea pig, Abcam, MA), glucagon (1:2000, mouse, Abcam), collagen I (1:500, rabbit, Abcam), α-smooth muscle (1:100, mouse, Sigma-Aldrich), iNOS(1:100, rabbit, Abcam), CD163 (1:200, mouse, Bio-rad, CA, USA), albumin(1:50, mouse, Proteintech, Wuhan, China), pro-surfactant protein C (SP-C, 1:50, rabbit, Proteintech), and CD206 (1:500, rabbit, Abcam) and were then incubated with Alexa Fluor 488/594-conjugated secondary antibodies (1:500, Invitrogen, USA) at room temperature for 2 h. Slides were observed under a laser scanning confocal microscope.

### Echocardiography

Rats were anaesthesized by isoflurane inhalation. Echocardiography (VisualSonics VEVO 2100, Toronto, ON, Canada) was performed by the same cardiologist who was blind to the treatments.

### Western blot analysis

Total protein was extracted from samples of epididymal adipose tissue, and the procedure was carried out as described previously. The primary antibodies were PI3K (1:1000, rabbit, Cell Signaling Technology, MA, USA), total or phosphorylated AKT (Ser473) (p-AKT) (1:1000, rabbit, Cell Signaling Technology), and β-actin (1:2000, mouse, ZSGB-Bio, Beijing, China). The secondary antibodies were goat anti-rabbit and rabbit anti-mouse IgG horseradish peroxidase (HRP) from the ZSGB-Bio company. The blots were analysed using ImageJ software (National Institutes of Health, Bethesda, MD).

### Quantitative real-time PCR

Total RNA was isolated from rat adipose tissue, liver tissue, renal tissue, and lung tissue using Trizol reagent (Life technologies, Frederick, USA) and was reverse-transcribed with a reverse transcription kit (Thermo Scientific, CA, USA)) according to the manufacturer’s instructions. Real-time polymerase chain reaction (RT-PCR) was performed on ABI Prism thermal cycler model StepOnePlus (Applied Biosystems, CA, USA) using a SYBR Green PCR master mix (Applied Biosystems). The thermal cycling programme was 94 °C for 3 min, followed by 94 °C for 30 s, 60 °C for 30 s, and 72 °C for 30 s for 40 cycles. Melting curve analysis was included to ensure primer specificity. The primers are listed in Additional file 1: Table S1.

### Enzyme-linked immunosorbent assay

Serum from each group were collected and stored at − 80 °C until they were thawed for the assay. The concentrations of IL-1β, IL-6, IL-10, and TNF-α in serum were measured using ELISA kits from Multi Sciences LTD. (Hangzhou, China). The levels of serum insulin were assessed using ELISA kits from Elabscience (Wuhan, China). The levels of C-peptide were assessed using ELISA kits from Millipore (St. Charles, MO). All procedures were performed according to the manufacturer’s instructions.

### Statistical analysis

Data are presented as mean ± standard deviation (SD) for normally distributed data and as mean [interquartile range] when non-normally distributed. Normality was assessed using the one-sample Kolmogorov–Smirnov test. Statistical differences between two groups were analysed by either unpaired Student *t* test (normally distributed data) or Mann–Whitney *U* test (non-normally distributed data), and differences between multiple groups of data were assessed by one-way analysis of variance (ANOVA) with Bonferroni’s multiple comparison test. Statistical significance was defined as *p* < 0.05. All analyses were accomplished using the software in GraphPad Prism 3.0 (GraphPad Software, San Diego, CA, USA) and SPSS statistical software version 25 (SPSS Inc., IBM, USA).

## Results

### Establishment of the HFD/STZ-induced long-term T2DM rat model

**Fig. 1:**
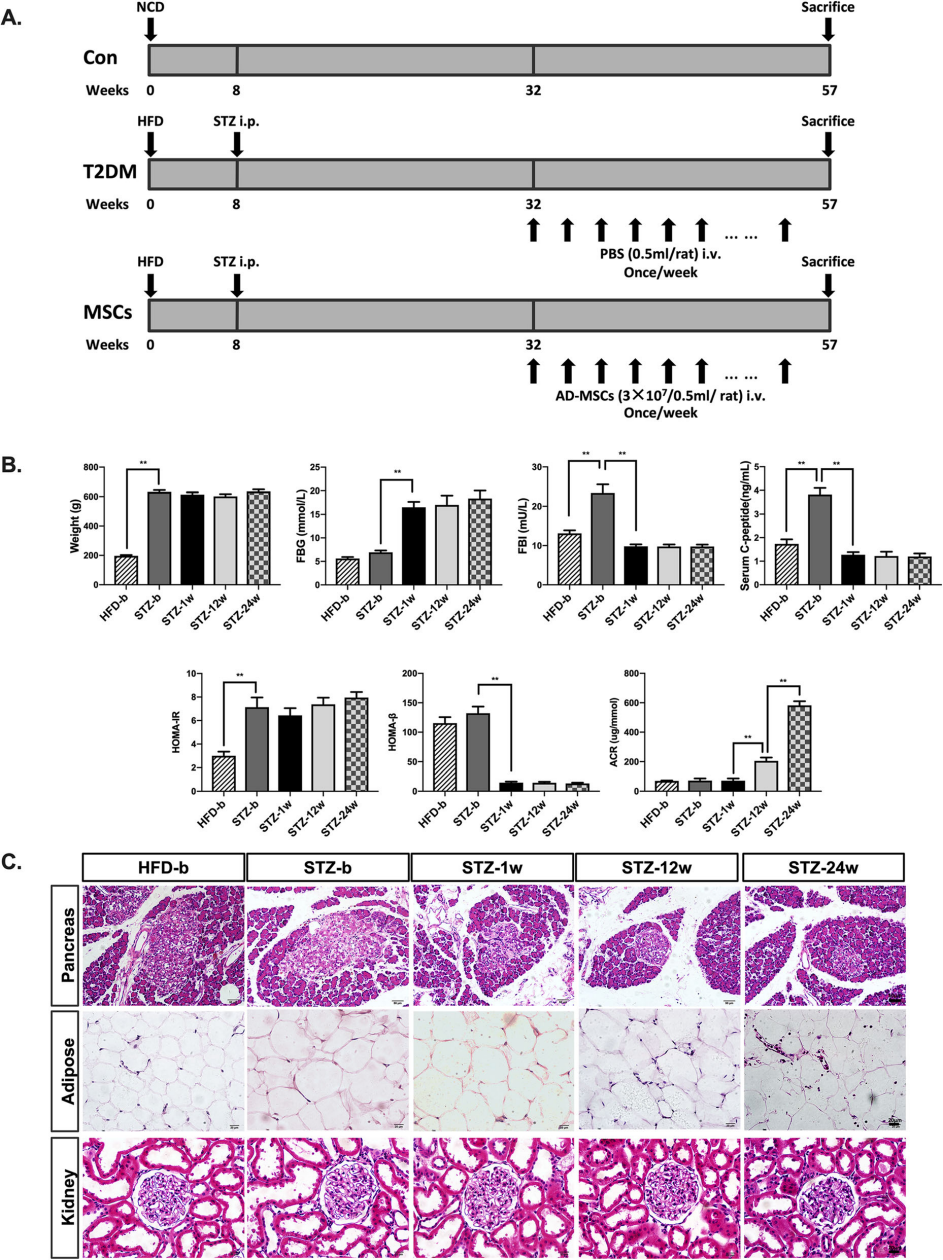
Establishment of the HFD/STZ-induced long-term T2DM rat model. **a** Illustration for the study design. To produce the long-term T2DM complication rodent model, 8-week-old male SD rats were fed a HFD for 8 weeks, followed by an STZ injection at a single dose of 25 mg/kg. HFD feeding and hyperglycaemia were maintained in the newly diabetic rats for 24 weeks. Then, the long-term T2DM complication rats were randomly treated with one of the following interventions: infusions of 3 × 10^6^ ADSCs suspended in 0.5 ml of PBS through the tail vein once a week for 24 weeks (referred to as the MSC-treated group, *N* = 6) or infusions of 0.5 ml PBS alone once a week for 24 weeks (referred to as the T2DM group, *N* = 6). Normal rats of the same age that fed NCD were used as the control (referred to as the control group, *N* = 6). On week 57 (after 24 times of ADSC treatment), the therapeutic effects of ADSCs on T2DM complications were assessed. **b** Body weight, FBG, FBI, serum C-peptide, HOMA-IR, HOMA-β, and ACR at the time point before HFD (HFD-b), before STZ injection (STZ-b), 1 week after STZ injection (STZ-1w), 12 weeks after STZ injection (STZ-12w), and 24 weeks after STZ injection (STZ-24w). **c** Representative sections of epididymal adipose, pancreas, and kidney staining with H&E; bars = 50, 50, and 20 μm. *N* = 6 rats per group; *p < 0.05, **p < 0.01

The long-term T2DM complication rat model was induced by a HFD combined with a low-dose STZ (Fig. 1a). Eight-week-old male SD rats were provided with a HFD for 8 weeks. After that, a single dose of 25 mg/kg STZ was intraperitoneally injected. Random glucose was consecutively measured, and rats with more than three random glucose level measurements ≥ 16.7 mmol/l were considered diabetic. Then, the T2DM rats were fed with a HFD for 24 more weeks. Eight weeks’ HFD resulted in over 400-g gain of weight. In morphology, the average size of adipocytes was much larger. In addition, HOMA-IR and the levels of FBI and C-peptide significantly increased, all suggesting the presence of insulin resistance. At 1 week after STZ injection, the rats showed hyperglycaemia concomitant with decreases in FBI and C-peptide levels. Histological analysis showed morphological destruction of pancreatic islets and HOMA-β decreased from 132.2 to 14.6. No further β cell function deterioration was detected afterwards. To evaluate the stage of diabetic complications, we chose the kidney for an example. At 12 weeks after STZ injection, there was a mild increase in ACR. However, no significant changes of glomeruli could be found in histological analysis. At 24 weeks post-STZ injection, the level of ACR significantly increased by more than seven folds and H&E staining showed glomerular sclerosis, confirming the success of establishment of a long-term T2DM complication model (Fig. 1b, c).

### Multiple ADSC infusions improved glucose homeostasis by improving insulin sensitivity and promoting pancreatic islet recovery in long-term T2DM complication rats

**Fig. 2:**
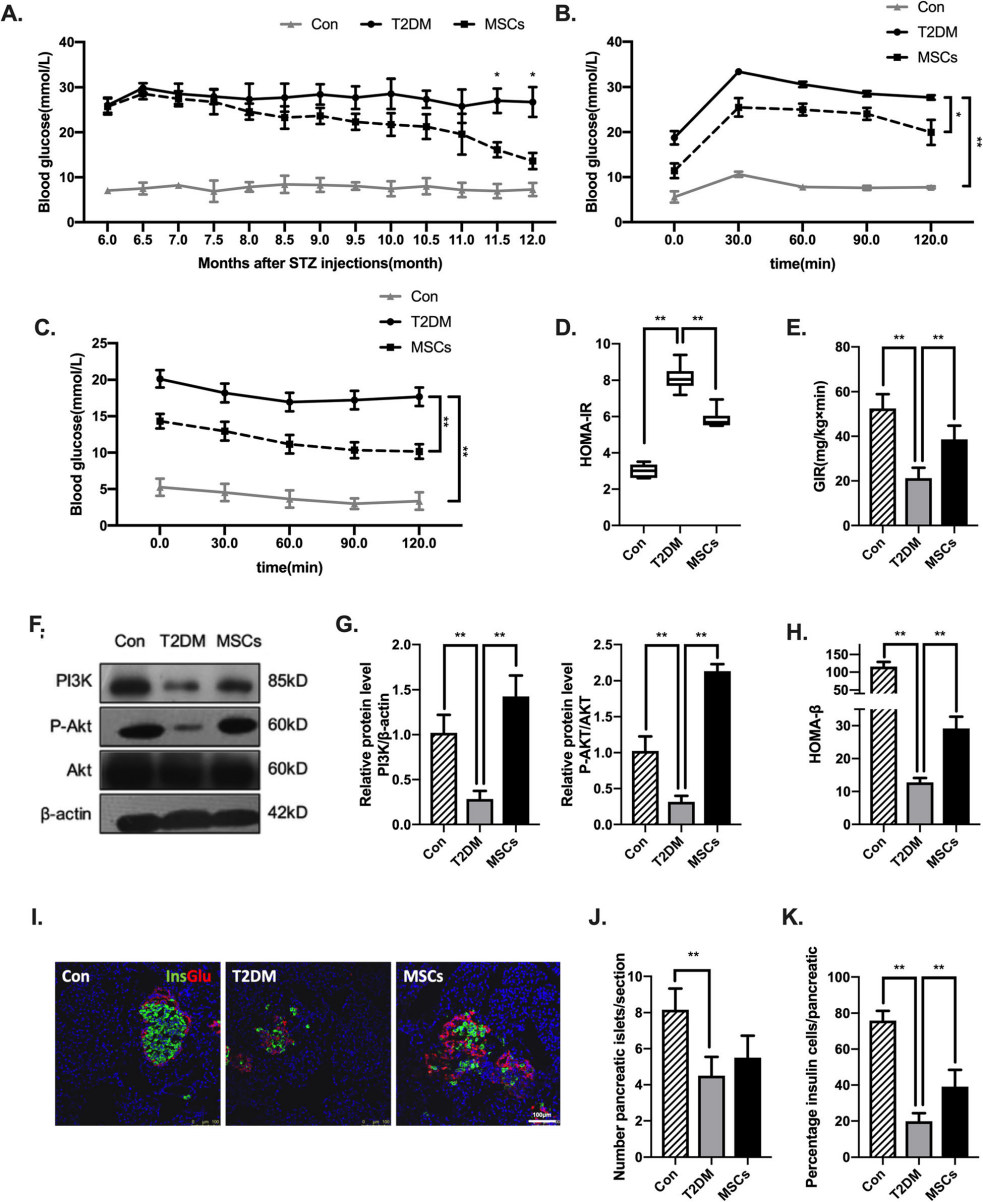
Multiple ADSC infusions improved glucose homeostasis by improving insulin sensitivity and promoting pancreatic islet recovery in long-term T2DM complication rats. **a** Blood glucose levels were detected consecutively after MSC infusions. **b**, **c** Concentration of glucose in the blood of three groups after a glucose tolerance test (intraperitoneal glucose tolerance test [IPGTT], **b**) or insulin tolerance test (intraperitoneal insulin tolerance test [IPITT], **c**). **d** HOMA-IR of each group. **e** GIR during hyperinsulinaemic-euglycaemic clamp analysis of each group. **f**, **g** Immunoblotting analysis of PI3K, p-AKT, and total AKT in epididymal adipose tissue; representative of three independent experiments. The ratios of PI3K to β-actin and p-AKT to total AKT were quantitated. **h** HOMA-β of each group. **i** The presence and distribution of insulin- (green) and glucagon-producing (red) cells were evaluated. Bars = 100 μm. **j**, **k** The β cell mass and percentage of β cells in the pancreatic islets were measured. *N* = 6 rats per group; *p < 0.05, **p < 0.01

The T2DM rats were randomly treated with one of the following interventions: ADSC infusions via the tail vein once a week for 24 weeks (referred to as the MSC-treated group) or PBS infusions once a week for 24 weeks (referred to as the T2DM group). Normal rats of the same age that fed NCD were used as the control (referred to as the control group) (Fig. 1a). During the first 12 weeks of MSC infusions, there was no significant decrease in random blood glucose levels in the MSC-treated group. However, 4 weeks later, the MSC-treated group started to show a persistent and gradual decrease in blood glucose level, while the T2DM group showed persistent hyperglycaemia (Fig. 2a). To measure glucose clearance efficacy and insulin sensitivity, intraperitoneal IPGTTs, IPITTs, and hyperinsulinaemic–euglycaemic clamp studies were performed 1 week after the 24th treatment. The results of the IPGTT revealed greatly improved glucose clearance in the MSC-treated group (Fig. 2b). Improvements in the IPITT, homeostatic model assessment of insulin resistance (HOMA-IR), and glucose infusion rate evidenced by the hyperinsulinaemic–euglycaemic clamp study and analysis of PI3K and P-AKT expression in adipose tissue (Fig. 2c–g) indicated a marked enhancement in insulin sensitivity after the MSC infusions. In addition, the MSC infusions markedly restored pancreatic islet function, as evidenced by the improvement in the homeostatic model assessment of β cell function (HOMA-β) (Fig. 2h). There was no significant increase in the number of islets, but the ratio of insulin-positive cells per islet was significantly increased compared with the same parameters in the untreated T2DM rats; these characteristics were identified by immunofluorescence analyses (Fig. 2i–k).

### Multiple ADSC infusions attenuated T2DM-induced kidney damage in long-term T2DM complication rats

**Fig. 3:**
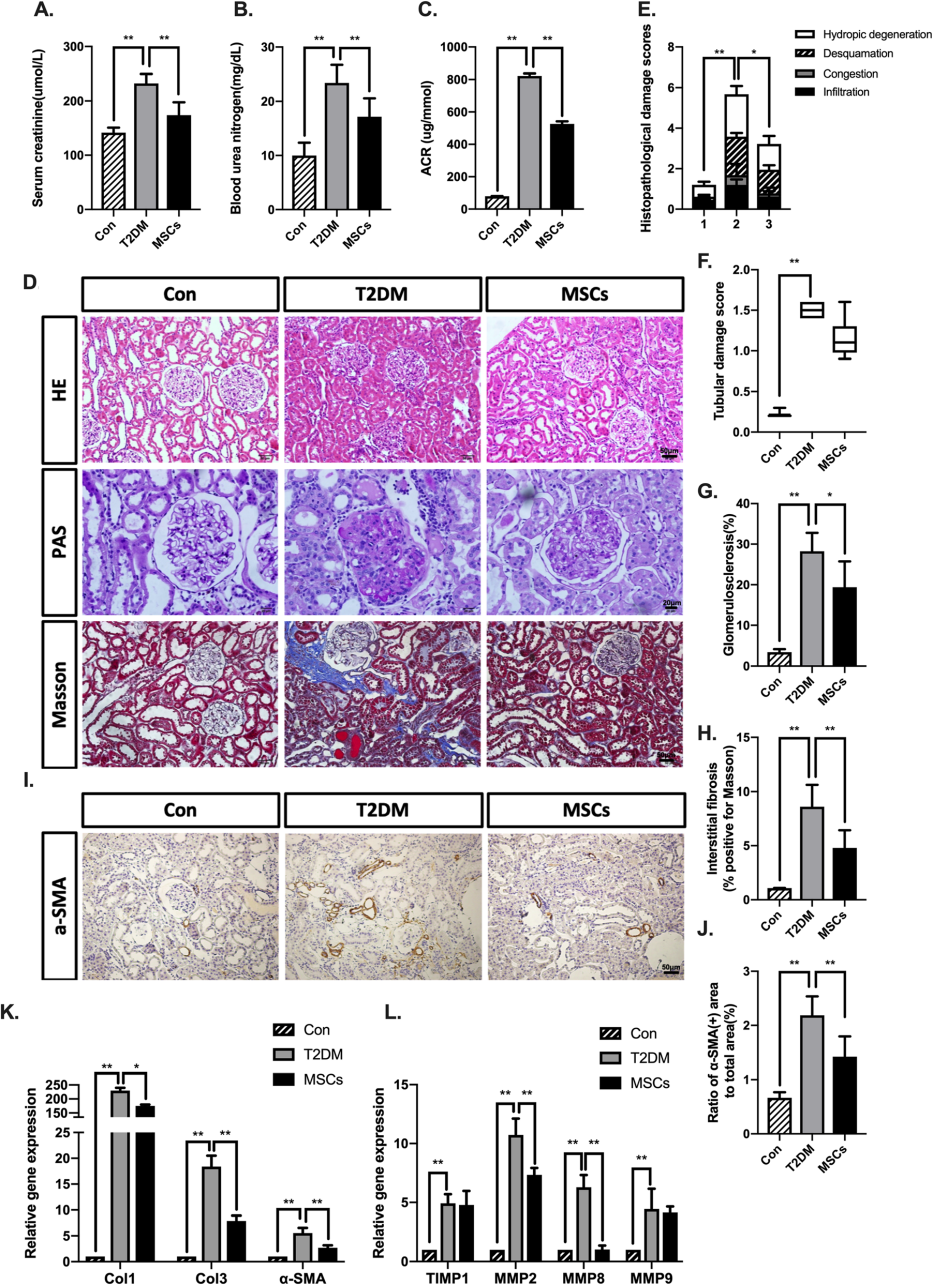
Multiple ADSC infusions attenuated kidney damage in long-term T2DM complication rats. **a**–**c** Serum creatinine, blood urea nitrogen, and urine ACR of each group were evaluated. **d** Representative kidney sections staining with H&E, PAS, and Masson’s trichrome; bars = 50, 20, and 50 μm. **e**, **f** Histopathological damage scores and tubular damage score were graded using H&E-stained sections. **g** Glomerulosclerotic injury was graded using PAS-stained sections. **h** Interstitial fibrosis was graded using Masson-stained sections. **i**, **j** Immunohistochemical staining and quantification of α-SMA area, bars = 50 μm. **k**, **l** Quantitative reverse transcriptase polymerase chain reaction analysis of gene expression in kidney tissue from control, T2DM, and MSC groups. Results are presented relative to those of control rats, set as 1. *N* = 6 rats per group; *p < 0.05, **p < 0.01

DN is a common vasculature complication in type 2 diabetic patients. Thus, we determined whether MSC infusions could reverse the progression of DN. As expected, the T2DM rats presented with deteriorated kidney function; serum creatinine reached 233.8 μmol/l, blood urea nitrogen (BUN) reached 23.3 mg/dl, and the urinary ACR reached 814.2 μg/mmol, which was more than ten folds higher than the level of the control group. MSC treatment significantly attenuated these indexes, demonstrating improved kidney function (Fig. 3a–c). H&E, PAS, and Masson’s trichrome staining showed obvious infiltration, swelling of tubule cells, hypertrophy of glomeruli, glomerulosclerotic changes, and tubulointerstitial fibrosis in T2DM rats, which were markedly reversed by MSC treatment (Fig. 3d–h). To verify the amelioration of renal fibrosis by MSC treatment, we further examined the expression of alpha smooth muscle actin (α-SMA) at the protein level in all the groups. The results indicated that the expression level of α-SMA was markedly increased in T2DM rats compared to that in the control group, whereas the percentage of α-SMA in the MSC-treated rats decreased to approximately 64.3% of that of the T2DM rats (Fig. 3i, j). Gene expression quantification showed that several tissue fibrosis markers, such as collagen type I (col1), collagen type III (col3), α-SMA, and matrix metalloproteinase (MMP)-2 and MMP-8 were markedly upregulated in the T2DM rats but were significantly downregulated in the MSC-treated rats (Fig. 3k, l).

### Multiple ADSC infusions attenuated T2DM-induced liver disease in long-term T2DM complication rats

**Fig. 4:**
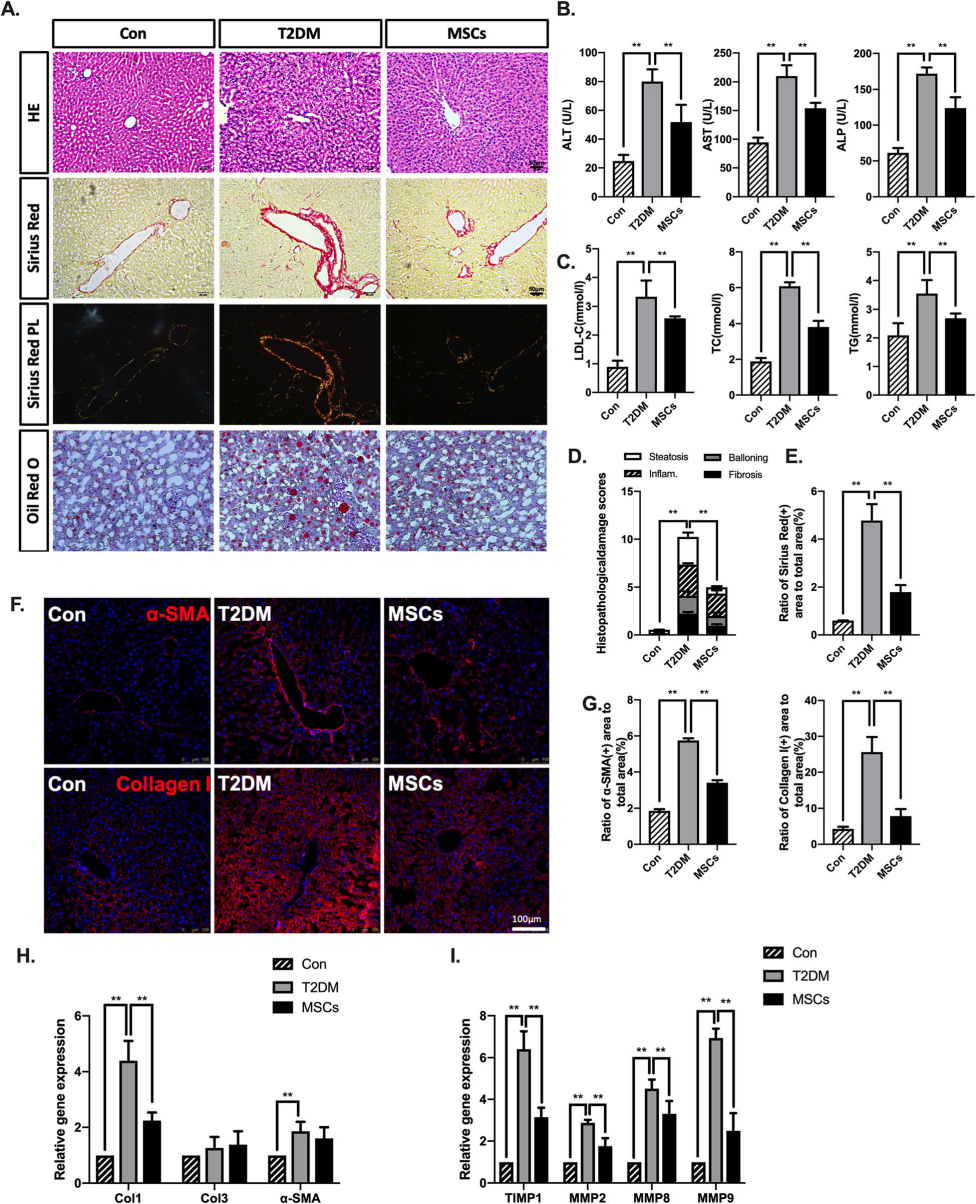
Multiple ADSC infusions attenuated liver disease in long-term T2DM complication rats. **a** Representative liver sections staining with H&E, Sirius Red, and Oil Red O; bars = 50, 50, 50, and 20 μm. **b** Levels of serum ALT, AST, and ALP of each group were evaluated. **c** Levels of serum LDL-C, TC, and TG of each group were evaluated. **d** Histopathological damage scores were graded using H&E-stained sections. **e** Ratio of Sirius Red-positive area to total area. **f**, **g** Immunofluorescence and quantification of α-SMA and collagen I area; bars = 100 μm. **h**, **i** Quantitative reverse transcriptase polymerase chain reaction analysis of gene expression in liver tissue from control, T2DM, and MSC groups; results are presented relative to those of control rats, set as 1. *N* = 6 rats per group; *p < 0.05, **p < 0.01

Patients with advanced T2DM are always affected by nonalcoholic fatty liver disease (NAFLD) and dyslipidaemia. Therefore, we further examined the histopathological changes and important serum indicators related to hepatic function and lipid metabolism. The T2DM rats presented NASH-like features such as steatosis and inflammation assessed by the histopathological evaluation of the NASH score, but these features were markedly attenuated in the MSC-treated group (Fig. 4a, d). The levels of serum liver enzymes and circulating TC, TGs, and LDL-C increased two- to fourfold in the T2DM rats compared to those in the normal control rats, while the MSC infusions substantially reversed this trend (Fig. 4b, c). Positive Sirius Red staining together with expression of α-SMA and collagen I, which were readily seen in the T2DM rats and highlighted a significant increase in liver fibrosis, were rare in the MSC-treated group (Fig. 4a, e–g). Moreover, treatment with MSCs significantly decreased col1 transcripts, with beneficial trends of profibrogenic gene expression of tissue inhibitor of metalloproteinases-1 (TIMP-1) and MMP-2, 8, and 9 (Fig. 4h, i), confirming that MSC infusions significantly attenuated T2DM-induced liver disease.

### Multiple ADSC infusions attenuated T2DM-induced lung disease in long-term T2DM complication rats

**Fig. 5:**
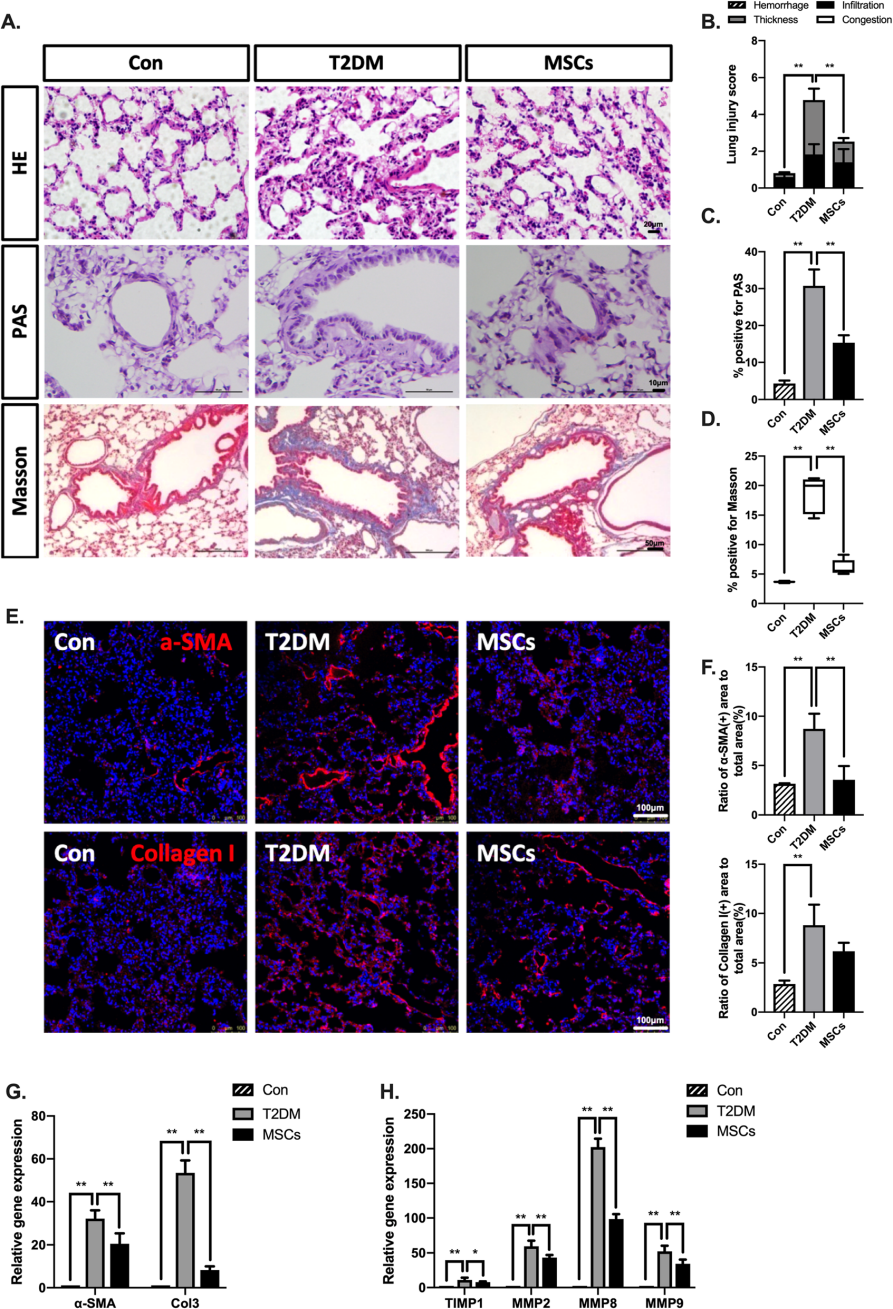
Multiple ADSC infusions attenuated lung disease in long-term T2DM complication rats. **a** Representative lung section staining with H&E, PAS, and Masson’s trichrome; bars = 20, 10, and 50 μm. **b** Lung injury scores were graded using H&E-stained sections. **c** Ratio of PAS staining-positive area to total area. **d** Ratio of Masson staining-positive area to total area. **e**, **f** Immunofluorescence and quantification of α-SMA and collagen I area; bars = 100 μm. **g**, **h** Quantitative reverse transcriptase polymerase chain reaction analysis of gene expression in lung tissue from control, T2DM, and MSC groups; results are presented relative to those of control rats, set as 1. *N* = 6 rats per group; *p < 0.05, **p < 0.01

An increasing number of studies indicating physiological and structural abnormalities in the lungs of both type 1 and type 2 diabetic patients suggests that the lungs should be considered a ‘target organ’ [30]. The photomicrograph analyses of lung tissue from the T2DM group showed a significant increase in alveolar thickness, infiltration of inflammatory cells, and disordered lung tissue structure, but these histopathological changes were diminished in the MSC-treated group (Fig. 5a). The lung injury score of the T2DM group was significantly higher than the score of the MSC-treated group (Fig. 5b). In addition, in the results of the PAS staining, the lungs of the T2DM rats showed more glycogen granules than those of the normal control rats, while treatment with MSCs significantly reduced the positive PAS-stained area to 48.7% of that of the T2DM group (Fig. 5c). Pulmonary fibrosis was detected in the lung sections stained with Masson’s trichrome staining and is expressed as the percentage of the total area stained for collagen. Treatment with MSCs decreased the total amount of collagen in the alveolar space back to normal levels (Fig. 5d). These results were consistent with the trends of α-SMA expression evidenced by immunofluorescence staining and the relative RNA expression of col3, α-SMA, TIMP-1, and MMP-2, 8, and 9 (Fig. 5e–h).

### Multiple ADSC infusions attenuated T2DM-induced cardiac changes in long-term T2DM complication rats

**Fig. 6:**
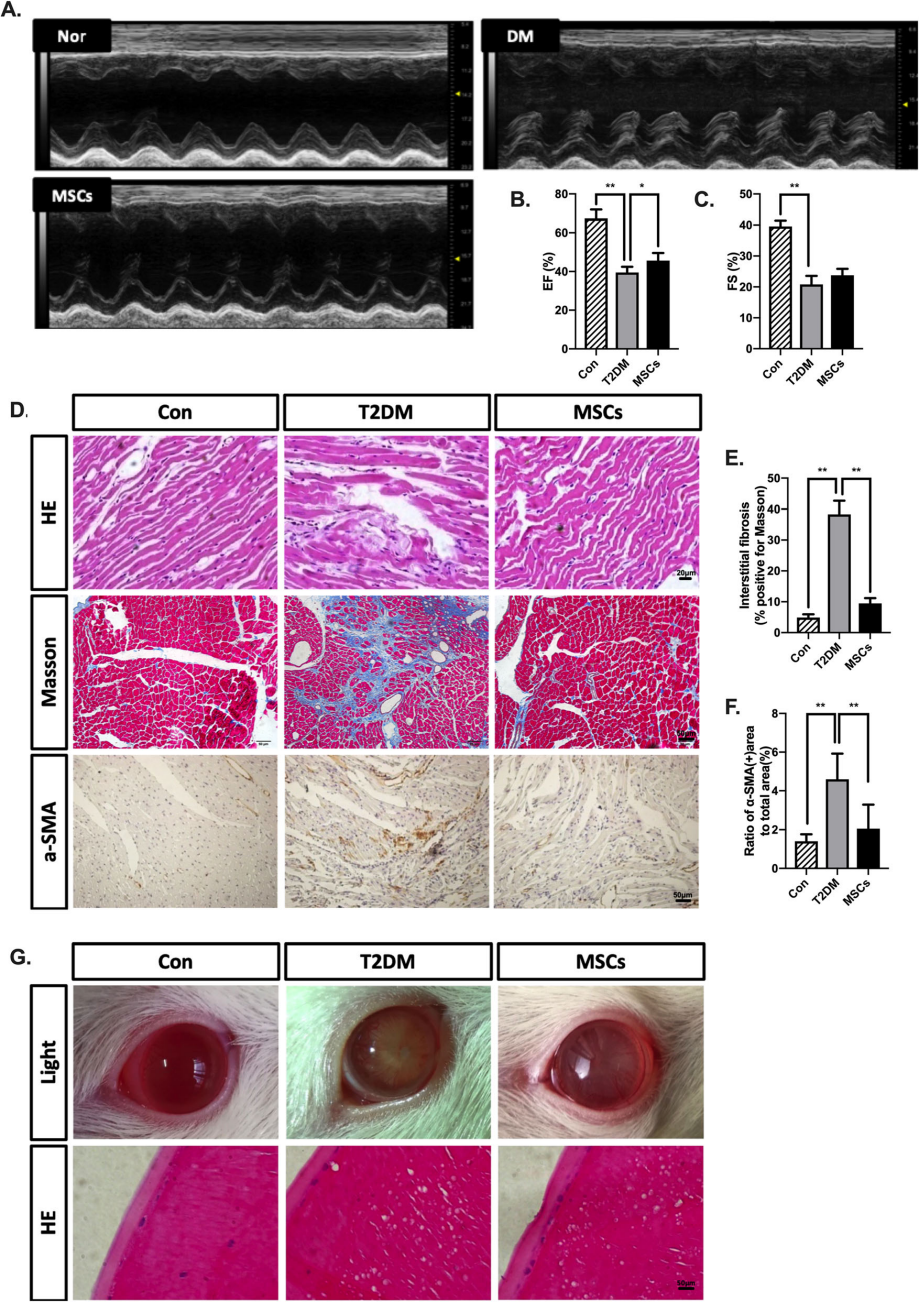
Multiple ADSC infusions attenuated cardiac changes and exerted an anti-cataract effect in long-term T2DM complication rats. **a**–**c** Representative M-mode echocardiograms and quantification of left ventricular ejection fraction (EF) and fractional shortening (FS). **d** Representative cardiac sections staining with H&E, Masson’s trichrome, and immunohistochemical staining of α-SMA; bars = 20, 50, and 50 μm. **e** Interstitial fibrosis was graded using Masson-stained sections. **f** Quantification of α-SMA area. **g** Representative photographs via the camera and representative sections staining with H&E; bars = 50 μm. *N* = 6 rats per group; *p < 0.05, **p < 0.01

The functional cardiac consequences of diabetes and MSC treatment were evaluated using echocardiography. Ejection fractions and fractional shortening in the T2DM rats were 39.37% and 20.49%, respectively, which were significantly lower than those of the control rats; however, MSC treatment improved ejection fractions (Fig. 6a–c). As evidenced by the H&E staining results, the diameter of the cardiomyocytes was significantly larger in the T2DM group than that in the control group. In turn, the MSC treatment alleviated the diabetes-induced cardiomyocyte hypertrophy in the T2DM rats. Additionally, the cardiac fibrosis assessment involved Masson’s trichrome staining and the expression of α-SMA. Obvious fibrosis in the heart, as well as a destroyed and disorganized collagen network structure, was observed in the T2DM rats. However, the fibrotic changes in the heart were significantly mitigated by MSC treatment. In addition, compared with the normal control rats, the T2DM rats showed increased cardiac expression levels of α-SMA, while the MSC treatment significantly attenuated these changes (Fig. 6d–f), indicating that MSC treatment attenuated T2DM-induced cardiomyocyte hypertrophy and interstitial fibrosis.

### Multiple ADSC infusions exerted an anti-cataract effect in long-term T2DM complication rats

Cataracts are among the common complications of diabetes mellitus. As shown in Fig. 6g, the lenses of the normal rats remained clear and transparent without any turbidity. In contrast, the rats in the T2DM group presented with cloudy lenses with nuclear opacity. Most of the lenses of the MSC-treated rats were significantly clearer than those of the rats in the T2DM group. The H&E staining results also validated the anti-cataract effect of MSC infusions in vivo (Fig. 6g).

### UC-MSC tracking

Twenty-four hours after injection, among the organs examined, only a small number of ADSCs could be detected in the lung and liver. However, engraftment of ADSCs was barely seen in the pancreas, adipose tissue, myocardium, or kidney of T2DM rats. The trends were similar at 7 days after injection (Additional file 1: Figure S1a,b). In order to analyze whether the CM-Dil-positive ADSCs in the pancreas, liver, and lung could differentiate into β cell, hepatocyte, or pneumocyte of the alveolar epithelium, we separately labelled CM-Dil-positive cells with insulin, albumin (a marker of hepatocyte), and SP-C (a marker of pneumocyte). Results of immunofluorescence staining showed that most of the CM-Dil-positive cells in the pancreas, lung, and liver of T2DM rats did not express cellular marker of β cell, hepatocyte, or pneumocyte, indicating that ADSCs did not differentiate into those cellular types (Additional file 1: Figure S1c). Besides, the number of ADSC homing to the liver and lung can hardly explain the significant therapeutic effect on liver and lung disease. These results indicated that the effect of ADSCs on the treatment of diabetic complication does not depend on the homing of ADSCs into target organs.

### Multiple ADSC infusions attenuated inflammation and changed the phenotypes of macrophages in the target organs of diabetic complications in long-term T2DM complication rats

**Fig. 7:**
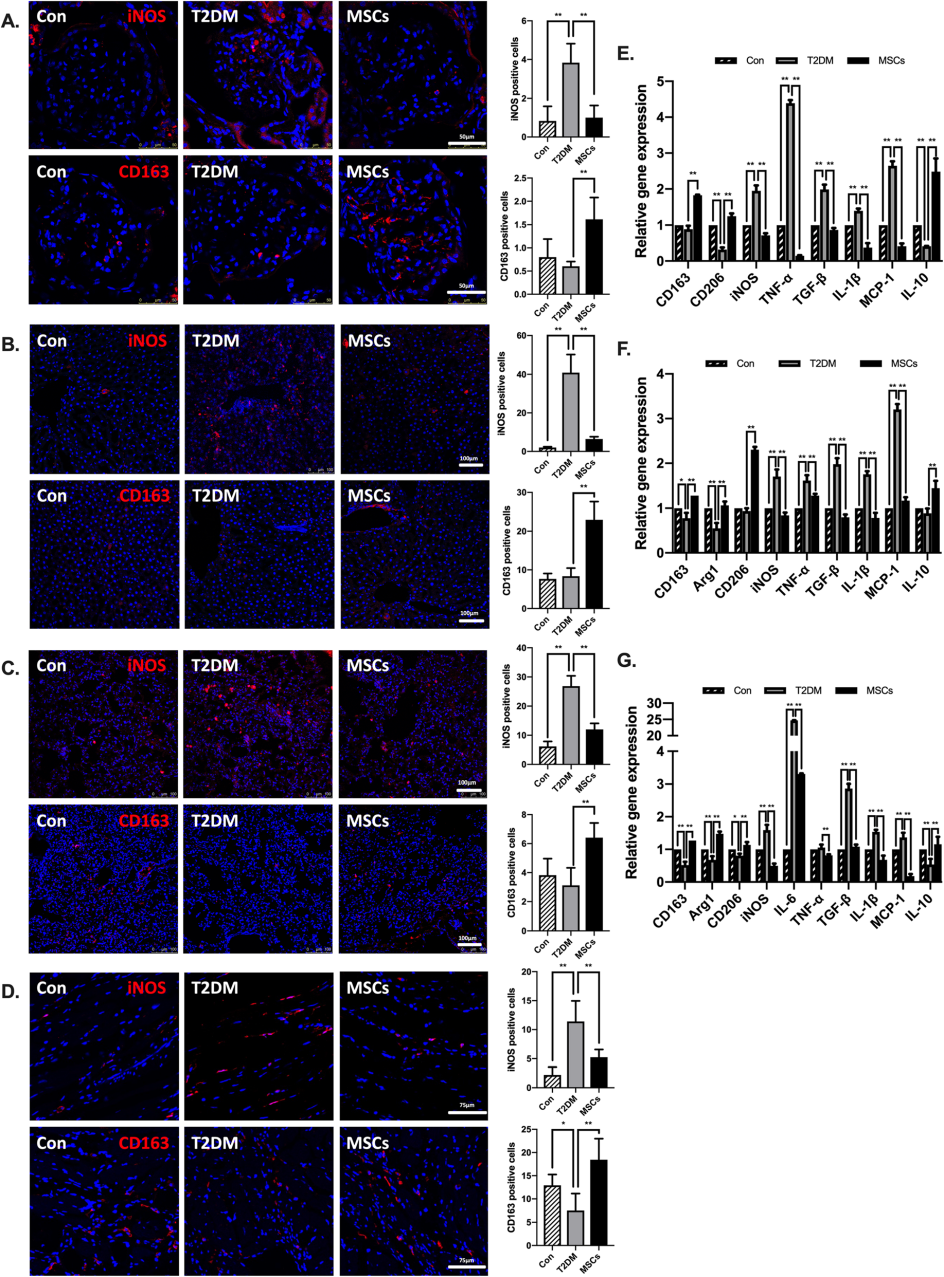
Multiple ADSC infusions attenuated inflammation and changed the phenotypes of macrophages in the target organs in long-term T2DM complication rats. **a** Representative of iNOS-positive or CD163-positive cells in kidney tissue by immunofluorescence and quantification of iNOS-positive or CD163-positive cells; bars = 50 μm. **b** Representative of iNOS-positive or CD163-positive cells in liver tissue by immunofluorescence and quantification of iNOS-positive or CD163-positive cells; bars = 100 μm. **c** Representative of CD11c-positive or CD163-positive cells in lung tissue by immunofluorescence and quantification of iNOS-positive or CD163-positive cells; bars = 100 μm. **d** Representative of iNOS-positive or CD163-positive cells in the myocardium by immunofluorescence and quantification of iNOS-positive or CD163-positive cells; bars = 75 μm. **e**–**g** Quantitative reverse transcriptase polymerase chain reaction analysis of gene expression in the kidney, liver, and lung tissue from control, T2DM, and MSC groups; results are presented relative to those of control rats, set as 1. *N* = 6 rats per group; *p < 0.05, **p < 0.01

**Fig. 8:**
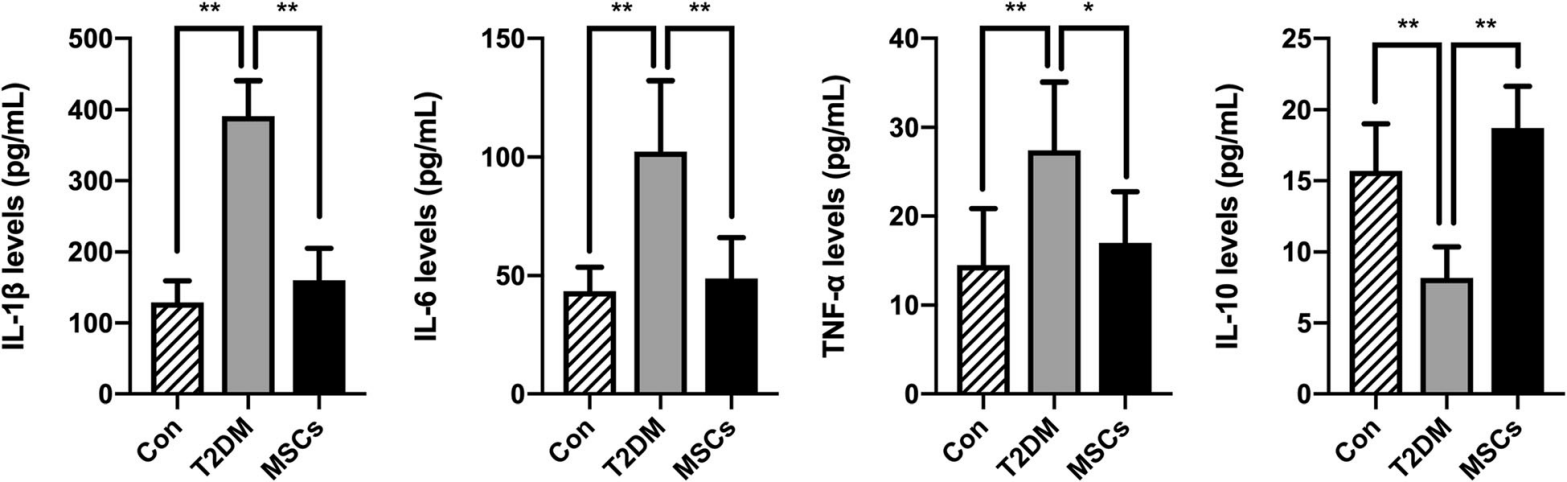
Changes of inflammatory cytokine in serum following multiple ADSC infusions. ELISA assays of IL-10, IL-6, IL-1β, and TNF-α in serum in control, T2DM, and MSC groups. Data are presented as the mean ± SD; *N* = 6 rats per group; *p < 0.05, **p < 0.01

The anti-diabetic effects of MSCs are suggested to correlate with their modulation of macrophages, so we further investigated the changes in the phenotypes of macrophages and the inflammatory state in the target organs of diabetic complications by analysing the kidney, hepatic, pulmonary, and cardiac tissues of the T2DM rats. M1 macrophages were identified by iNOS, and M2 macrophages were identified by CD163 and CD206. Confocal micrographs showed that the number of CD163-positive and CD206-positive cells was elevated in the MSC-treated group, while the number of iNOS-positive cells decreased in the MSC-treated group compared with the numbers in the T2DM group (Fig. 7a–d; Additional file 1: Figure S2a-d); there were no significant differences in the number of F4/80-positive cells (data not shown). Real-time polymerase chain reaction (PCR) analysis revealed a lower expression of genes encoding molecules typically linked to inflammation (TNF-α, IL-1β) or fibrosis (TGF-β) and a higher expression of genes encoding anti-inflammatory molecules (IL-10) in the MSC-treated group (Fig. 7e–g). We also observed lower expression of the gene encoding iNOS and higher expression of the genes encoding Arg1, CD206, and CD163 (Fig. 7e–g). The levels of inflammatory cytokines in the serum of each animal group revealed that ADSC infusion was able to not only inhibit pro-inflammatory cytokines IL-6, IL-1β, and TNF-α expression but also increase anti-inflammatory cytokine IL-10 systematically (Fig. 8). These results indicated that MSC infusions promoted M2 polarization and limited the expression of genes encoding pro-inflammatory molecules in the T2DM rats.

## Discussion

It has been indicated that MSCs have potential as a regenerative therapy for diabetes-associated complications (for recent review, see [31, 32]). Most current studies have focused on the prevention or amelioration of early-stage diabetic complications. However, the therapeutic effects of MSCs in reversing the long-term diabetic complications have not been reported. In our study, the therapeutic effects of MSCs are particularly understudied in diabetic animals with a disease duration of more than 24 weeks. The present data suggested that multiple MSC infusions not only effectively restored glucose homeostasis and alleviated insulin resistance but also ameliorated hyperlipidaemia and altered the progression of long-term diabetic complications, such as CKD, NASH, lung fibrosis, and cataracts, and improved cardiac function in T2DM rats.

Animal models are a key resource to explore the pathogenesis of diabetic complications and reduce the gap between preclinical and clinical research. Although genetically (spontaneously) modified animal models, such as db/db mice or Zucker diabetic fatty (ZDF) rats, present with many of the metabolic and organic failures that occur in T2DM [33–35], the development of experimentally (non-spontaneously) induced diabetic animals can further help to mimic the different stages of T2DM progression. We developed and characterized a rat model of long-term T2DM complications that went through three stages of T2DM development: (i) the onset of obesity with insulin resistance and compensatory hyperinsulinaemia without hyperglycaemia representing a large fraction of prediabetes, (ii) a short duration of T2DM with hyperglycaemia and relative hypoinsulinaemia characterized by a decline in the secretory capacity of the pancreatic β cells, and (iii) a long duration of T2DM with significant signs of dyslipidaemia, hepatic fibrosis, and steatosis, as well as cardiac and renal dysfunction. Altogether, this alternative model is easy to generate at a relatively low cost, widely available, and useful for exploring behavioural or drug testing related to long-term diabetic complications and for studying the pathogenesis of diabetic complications, such as diabetic cardiomyopathy and nephropathy.

Regarding the treatment of T2DM, the ability of MSCs to ameliorate circulating glucose levels may be short-lasting. Our previous study showed that in T2DM rats, a single MSC infusion alleviated hyperglycaemia for only 1 week [36]; then, the hyperglycaemia steadily returned to the pretreatment values. However, hyperglycaemia decreased to approximately normal levels after at least three infusions. Moreover, compared to early-phase (1 week) T2DM rats, late-phase (5 week) T2DM rats exhibited slower and poorer effects after the MSC infusions [37]. Thus far, it has been shown that hyperglycaemia in T2DM can be reversed more effectively with multiple MSC infusions. The onset of the treatment effect may be later considering the longer course of T2DM. The present study showed that the blood glucose levels of the T2DM rats exhibited a persistent and gradual decrease until the 16th MSC infusion, and after 24 MSC treatments, the hyperglycaemia decreased to approximately normal levels. The results of this study suggest that a multiple MSC infusion strategy, rather than a single infusion or several infusions, offers a superior clinical option for advanced T2DM patients.

For the first time, we infused MSCs in long-term T2DM complication rats once a week for 24 weeks, which resulted in desirable treatment effects. However, some studies have illustrated the potential mechanisms of MSC immunosuppression, and one major mechanism is that MSCs suppress T cell proliferation due to their effects on soluble factors [38] such as IL-10 [39], which was identified at a significantly higher level in our study. Taking these ideas into account, it is necessary to perform animal experiments before developing a clinical treatment to determine whether multiple infusions of MSCs are safe for recipients and whether the body’s normal immunity is suppressed after the MSC treatments. After infusing ADSCs into the rats, there was no significant effect on food intake or body weight, and the treatment did not induce anaphylaxis or altered leukocyte concentration. These findings indicate that multiple intravenous infusions of MSCs have no obvious toxic effects on liver or kidney function or T cell immune suppression in T2DM rats.

Tissue macrophages are crucial players in T2DM-associated inflammation, which is characterized by an increased abundance of macrophages in different tissues along with the production of inflammatory cytokines [40]. Macrophages exhibit a phenotypic range between two extremes, M1 macrophages (pro-inflammatory) and M2 macrophages (anti-inflammatory). The salient finding of this study is that the MSC infusions suppressed renal, hepatic, pulmonary, and cardiac inflammation, which was accompanied by improvements of long-term complications and decreases in tissue fibrosis in the diabetic rats. According to our previous study, we found that MSCs alleviate insulin resistance and restore islet β cells by differentiating macrophages into the anti-inflammatory phenotype [41, 42]. The present results showed an increase in the M2 to M1 ratio with no significant changes in the number of F4/80(+) macrophages. These results suggest that instead of specifically fixing one target organ, MSCs systematically attenuated inflammation, and the anti-inflammatory actions involved the induction of the phenotypic switch from M1 macrophages to M2 macrophages rather than eliminating the recruitment of macrophages into renal, hepatic, pulmonary, and cardiac tissues.

However, how does increasing the M2 to M1 ratio promote recovery from long-term diabetic complications?

One possibility is that M2 macrophages antagonize the functions of the M1 macrophages that exacerbate tissue damage. The M2 macrophages are often linked with tissue repair because they can antagonize the functions of M1 macrophages that exacerbate tissue damage [43, 44]. The secretion of the inflammatory cytokine IL-1β by M1 macrophages has been shown to be a major driver of persistent inflammation and the pathogenesis of diabetes, atherosclerosis, and sterile inflammation [45, 46]. This finding is consistent with our results that revealed that the level of IL-1β in the target organs of the T2DM group was high, while the level decreased after MSC treatment. Another possible explanation is the anti-fibrotic roles of M2 macrophages. Tissue fibrosis is a sign of irreversible damage in age-related diseases and diabetic complications. Recent studies have suggested that M2 macrophages can also exhibit potent anti-fibrotic activity, particularly when the tissue-repair response becomes chronic. Indeed, mechanistic studies investigating the role of M2 macrophages in chronic models of fibrosis and cancer have suggested that M2 macrophages slow the suppression of local CD4+ T cell responses and reduce ECM production by myofibroblasts [47]. Furthermore, the nutrient competition between macrophages and neighbouring myofibroblast has been identified as an additional potent anti-fibrotic mechanism [48]. Our results indicated that MSC treatment resulted in improvements in inflammation, which were paralleled by a reduction in ECM deposition in renal, hepatic, pulmonary, and cardiac tissues. Overall, the increased M2 macrophage levels in various target organs may assist, enhance, or at least partly explain the therapeutic effects of MSCs on long-term T2DM complications.

A limitation of our study is that we did not directly test whether macrophage activation was necessary for the MSC-mediated protective effects. Because macrophage depletion alone delays the onset of diabetes [49] and attenuates diabetic complications, it may be difficult to determine whether macrophage depletion abrogated the therapeutic effects of MSCs on the long-term diabetic complications that occurred in our experimental setting. Hence, future experiments will need to address this issue.

## Conclusion

Multiple intravenous infusions of MSCs produced significant anti-diabetic effects. Moreover, MSCs attenuated systemic inflammation and altered the tissue M1/M2 ratio. These actions might be related to the alterations in the progression of long-term diabetic complications especially tissue fibrosis that leads to diseases, such as lung, liver, kidney, and cardiovascular complications, demonstrating the therapeutic potential of MSCs for long-term diabetic complications. These results can not only provide novel insights into intervention methods and therapeutic targets for the treatment of advanced stage of T2DM but may also provide crucial information related to other age-related diseases.

## Supplementary information

**Additional file 1: Table S1.** Primer sequences of target genes (rats). **Figure S1.** ADSCs’ homing efficiency in different tissues of long-term T2DM complications rats. ADSCs were CM-Dil (red) labelled in advance. After the infusion, T2DM rats were sacrificed at 24 h and 7 d. **a**, **b** Detection and quantification of ADSCs in adipose, pancreas, kidney, liver, lung, myocardium of long-termT2DM rats after transplantation; bars= 100μm. **c** The presence and distribution of CM-Dil (red) with insulin (green), SP-C (green) and albumin (green) were evaluated separately; bars= 100, 50, 50μm. **Figure S2.** Multiple ADSCs infusions induced an increase of M2 macrophages in the kidney, liver, lung and myocardium. **a** Representative of CD206 positive cells in kidney tissue by immunofluorescence; bars= 50μm. **b** Representative of CD206-positive cells in liver tissue by immunofluorescence; bars= 100μm. **c** Representative of CD206-positive cells in lung tissue by immunofluorescence; bars= 100μm. **d** Representative of CD206-positive cells in myocardium by immunofluorescence; bars= 75μm. N=6 rats per group, *, p<0.05; **, p<0.01.

## Data Availability

The datasets used and/or analysed during the current study are available.

## Abbreviations

MSCs: Mesenchymal stem cells
T2DM: Type 2 diabetes mellitus
DN: Diabetic nephropathy
STZ: Streptozotocin
HFD: High-fat diet
ADSCs: Adipose-derived mesenchymal stem cells
CKD: Chronic kidney disease
NASH: Nonalcoholic steatohepatitis
ESRD: End-stage renal disease
GvHD: Graft-versus-host disease
SLE: Systemic lupus erythaematosus
SD: Sprague–Dawley
FBS: Foetal bovine serum
PBS: Phosphate buffer solution
NCD: Normal chow diet
IPGTTs: Intraperitoneal glucose tolerance tests
IPITT: Insulin tolerance tests
FBI: Fasting blood insulin
FBG: Fasting blood glucose
TC: Total cholesterol
LDL-C: Low-density lipoprotein cholesterol
TG: Triglyceride
ALT: Alanine aminotransferase
AST: Aspartate aminotransferase
BUN: Blood urea nitrogen
ACR: Albumin to creatinine ratio
HOMA-IR: Homeostatic model assessment index of insulin resistance
HOMA-β: Homeostatic model assessment index of β cells
CM-Dil: Chloromethyl-benzamidodialkylcarbocyanine
α-SMA: α-Smooth muscle actin
EF: Ejection fractions
FS: Fractional shortening
TIMP-1: Tissue inhibitor of metalloproteinases-1
MMP: Matrix metalloproteinase
col1: Collagen type I
col3: Collagen type III
SP-C: Pro-surfactant protein C
