# Supplementary material for: Treatment with adipose tissue-derived mesenchymal stem cells exerts anti-diabetic effects, improves long-term complications, and attenuates inflammation in type 2 diabetic rats
Source: Stem Cell Res Ther. 2019 Nov 20;10:333. doi: 10.1186/s13287-019-1474-8 (PMC6868748; doi:10.1186/s13287-019-1474-8)
Supplement: Supplementary file 1 — Additional file 1: Table S1. Primer sequences of target genes (rats). Figure S1. ADSCs’ homing efficiency in different tissues of long-term T2DM complications rats. ADSCs were CM-Dil (red) labelled in advance. After the infusion, T2DM rats were sacrificed at 24 h and 7 d. a, b Detection and quantification of ADSCs in adipose, pancreas, kidney, liver, lung, myocardium of long-termT2DM rats after transplantation; bars= 100μm. c The presence and distribution of CM-Dil (red) with insulin (green), SP-C (green) and albumin (green) were evaluated separately; bars= 100, 50, 50μm. Figure S2. Multiple ADSCs infusions induced an increase of M2 macrophages in the kidney, liver, lung and myocardium. a Representative of CD206 positive cells in kidney tissue by immunofluorescence; bars= 50μm. b Representative of CD206-positive cells in liver tissue by immunofluorescence; bars= 100μm. c Representative of CD206-positive cells in lung tissue by immunofluorescence; bars= 100μm. d Representative of CD206-positive cells in myocardium by immunofluorescence; bars= 75μm. N=6 rats per group, *, p<0.05; **, p<0.01. [file 13287_2019_1474_MOESM1_ESM.pdf]

Additional file 1

Table S1 Primer sequences of target genes (rats)

Genes	Primer sequence (5'-3')	Product size (bp)	GeneBank Accession No.
β-actin	For: ACGGTCAGGTCATCACTATCG	155	NM_031144
	Rev: GGCATAGAGGTCTTTACGGATG		
iNOS	For: CCAACCTGCAGGTCTTCGATG	258	NM_012611
	Rev: GTCGATGCACAACTGGGTGAAC		
Arg1	For: CCAAGCCAAAGCCCATAGAG	59	NM_017134
	Rev: TCCTCGAGGCTGTCCCTTAG		
CD163	For: TGTAGTTCATCATCTTCGGTCC	98	NM_001107887
	Rev: CACCTACCAAGCGGAGTTGAC		
CD206	For: ACTGCGTGGTGATGAAAGG	68	NM_001106123
	Rev: TAACCCAGTGGTTGCTCACA		
MCP-1	For: CGTGCTGTCTCAGCCAGAT	71	NM_031530
	Rev: GGATCATCTTGCCAGTGAATG		
TNF-α	For: ATGGGCTCCCTCTCATCAGTTC	113	NM_012675
	Rev: CTCCTCCGCTTGGTGGTTTG		
IL-1β	For: TACCTATGTCTTGCCCGTGGAG	101	NM_031512
	Rev: ATCATCCCACGAGTCACAGAGG		
IL-10	For: ATGGCCCAGAAATCAAGGAGC	191	NM_012854
	Rev: GAAGATGTCAAACTCATTCATGGCC		

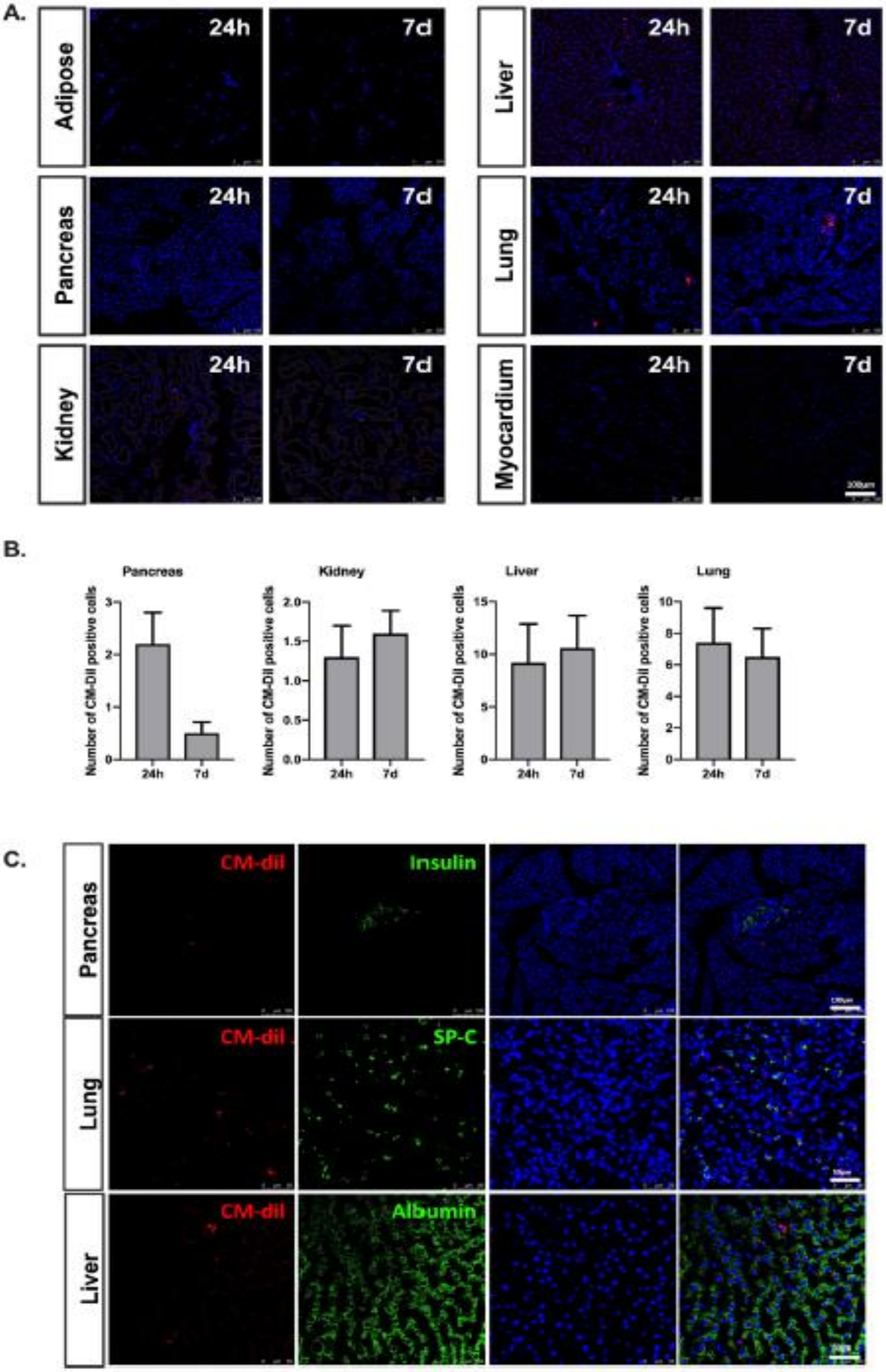

A.
Adipose
24h
7d
Liver
Pancreas
Lung
Kidney
Myocardium
200μm
B.
Pancreas
Kidney
Liver
Lung
Number of CM-DiI positive cells
24h
7d
C.
CM-dil
Insulin
SP-C
Albumin
Pancreas
Lung
Liver

Fig. S1. ADSCs' homing efficiency in different tissues of long-term T2DM complications rats. ADSCs were CM-Dil (red) labelled in advance. After the infusion, T2DM rats were sacrificed at 24 h and 7 d. **a,b** Detection and quantification of ADSCs in adipose, pancreas, kidney, liver, lung, myocardium of long-termT2DM rats after transplantation; bars= 100μm. **c** The presence and distribution of CM-Dil (red) with insulin (green), SP-C (green) and albumin (green) were evaluated separately; bars= 100, 50, 50μm.

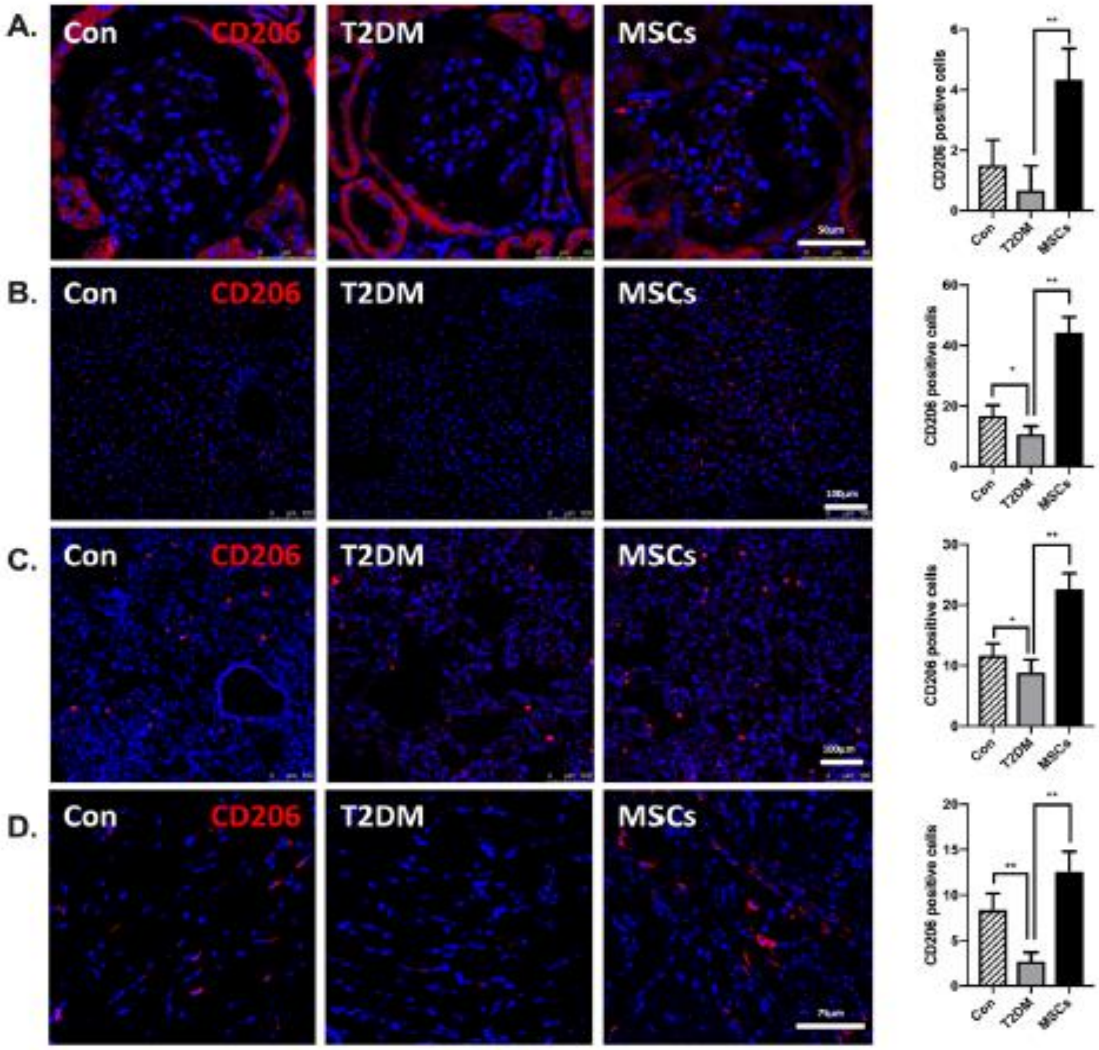

Fig. S2. Representative of CD206 positive cells in kidney tissue by immunofluorescence; bars= 50μm. **b** Representative of CD206-positive cells in liver tissue by immunofluorescence; bars= 100μm. **c** Representative of CD206-positive cells in lung tissue by immunofluorescence; bars= 100μm. **d** Representative of CD206-positive cells in myocardium by immunofluorescence; bars= 75μm. N=6 rats per group, *, $p<0.05$; **, $p<0.01$.
